# Enhanced Growth of Bacterial Cells in a Smart 3D Printed Bioreactor

**DOI:** 10.3390/mi14101829

**Published:** 2023-09-26

**Authors:** Eleftheria Maria Pechlivani, Sotirios Pemas, Alexandros Kanlis, Paraskevi Pechlivani, Spyros Petrakis, Athanasios Papadimitriou, Dimitrios Tzovaras, Konstantinos E. Hatzistergos

**Affiliations:** 1 Centre for Research and Technology Hellas, Information Technologies Institute, 6th km Charilaou-Thermi Road, 57001 Thessaloniki, Greece; sopemas@iti.gr (S.P.); alexkanlis@iti.gr (A.K.); athpapa@iti.gr (A.P.); dimitrios.tzovaras@iti.gr (D.T.); 2 Centre for Research and Technology Hellas, Institute of Applied Biosciences, 6th km Charilaou-Thermi Road, 57001 Thessaloniki, Greece; evi.pexli@gmail.com (P.P.); spetrak@certh.gr (S.P.); 3 Department of Genetics, Development and Molecular Biology, School of Biology, Faculty of Sciences, Aristotle University of Thessaloniki, 54124 Thessaloniki, Greece; kchatzistergos@bio.auth.gr

**Keywords:** additive manufacturing, 3D printing, bioreactor, IoT technologies, mobile user interface, bacterial growth, *Escherichia coli*

## Abstract

In the last decade, there has been a notable advancement in diverse bioreactor types catering to various applications. However, conventional bioreactors often exhibit bulkiness and high costs, making them less accessible to many researchers and laboratory facilities. In light of these challenges, this article aims to introduce and evaluate the development of a do-it-yourself (DIY) 3D printed smart bioreactor, offering a cost-effective and user-friendly solution for the proliferation of various bioentities, including bacteria and human organoids, among others. The customized bioreactor was fabricated under an ergonomic design and assembled with 3D printed mechanical parts combined with electronic components, under 3D printed housing. The 3D printed parts were designed using SOLIDWORKS^®^ CAD Software (2022 SP2.0 Professional version) and fabricated via the fused filament fabrication (FFF) technique. All parts were 3D printed with acrylonitrile butadiene styrene (ABS) in order for the bioreactor to be used under sterile conditions. The printed low-cost bioreactor integrates Internet-of-things (IoT) functionalities, since it provides the operator with the ability to change its operational parameters (sampling frequency, rotor speed, and duty cycle) remotely, via a user-friendly developed mobile application and to save the user history locally on the device. Using this bioreactor, which is adjusted to a standard commercial 12-well plate, proof of concept of a successful operation of the bioreactor during a 2-day culture of *Escherichia coli* bacteria (Mach1 strain) is presented. This study paves the way for more in-depth investigation of bacterial and various biological-entity growth cultures, utilizing 3D printing technology to create customized low-cost bioreactors.

## 1. Introduction

In recent decades, significant efforts have been dedicated to the development of various types of bioreactors [1,2,3]. It is widely recognized that when different organisms are subjected to specific conditions (e.g., temperature, CO_2_ percentage, rotational speed) within a continuously stirred bioreactor, they experience significant proliferation [4,5]. Bioreactors are containers where either intact cells or enzymes convert raw materials into biochemical products and potentially fewer unwanted by-products [2]. Bioreactors find applications in biology, biochemistry, and various laboratories, serving a multitude of purposes and offering promising capabilities for the growth and investigation of diverse biological entities, including bacteria, yeast, and cells [5]. By providing precise control over crucial factors such as temperature, pH, nutrient availability, oxygen levels and stirring speed, bioreactors enable researchers to establish optimal conditions for organism growth and proliferation, maximizing either biomass production or the synthesis of desired metabolites [2,6,7,8,9]. Overall, bioreactors play a crucial role in advancing biological research, serving as valuable tools for applications such as bacteria growth [10,11] and the generation of human organoids [12,13]. Consequently, they contribute significantly to the development of novel therapies and bio-based products [2,6,7].

To date, various types of bioreactors have been developed, including stirred-tank bioreactors [14], spinner flasks [15], perfusion bioreactors [16], and microfluidic bioreactors [17]. Each bioreactor has its unique advantages and limitations. Phelan et al. [18] summarized different types of bioreactors and their limitations. The referred types are the “Stirred tank” which has the disadvantages of the high volume, high shear and high cost, the “Clinostat/RWV”, designed by NASA, which also has the disadvantages of high volume, high cost and a tendency to be prone to failure and the “Hollow fiber” which is difficult to manufacturers. To overcome the challenges of significant size and high costs for Research and Development (R&D) applications, 3D printed bioreactors provide a smart and cost-effective solution for conducting experiments to test proof of concept in biology by utilizing smaller volumes of biological entities and develop various organisms [13,15] and bacterial cultures.

Various types of bioreactors are employed for bacterial growth in biological research, with each designed to cater to specific research requirements and objectives. The literature reveals a wide range of bioreactor types used for bacterial growth. For instance, the membrane bioreactor (MBR) [11] has been utilized for enriching anammox bacteria as free cells. Miller et al. [12] proposed a novel bioreactor suitable for microbial growth and productivity at extreme temperatures of up to 260 °C and pressures of up to 350 bar. Krustok et al. [19] investigated the growth dynamics of algal and bacterial cultures in photo-bioreactors. Boccazzi et al. [20] studied bacterial (*Escherichia coli*) cultures grown in rich or defined minimal media using a miniaturized 50 μL microbioreactor fabricated from polydimethylsiloxane (PDMS) and glass. These examples highlight the diverse types of bioreactors, each offering unique advantages in terms of agitation and mass transfer, depending on the bacterial culture, growth conditions, and experimental scale [21].

Current studies have shown two operational 3D printed bioreactors. The customized bioreactors are the SpinΩ [22] bioreactor and the Spinfinity (Spin∞) [13]. Qian et al. [22] developed the SpinΩ bioreactor using the 3D printing process for the parts and hardware that is accessible in the market for the purpose of generating forebrain, midbrain and hypothalamus organoids from human induced pluripotent stem cells (hiPSCs). Moreover, a more improved version of that bioreactor was developed by Romero-Morales et al. [13]. This improved 3D printed bioreactor called Spinfinity (Spin∞) has been developed to address the technical limitations of SpinΩ. SpinΩ had a number of drawbacks, including poor stability of certain components and an increased risk of contamination due to the device’s design [13]. Furthermore, a different type of low-cost 3D printed bioreactor is found in the literature, which was developed by Khan et al. [23], but it is a microfluidic bioreactor which has a more complicated design.

Although many attempts have been made to develop 3D printed bioreactors, none of these studies have presented the development of an Internet of things (IoT)-based bioreactor that also operates via a mobile application, thereby making it more user-friendly for potential users.

**Figure 1:**
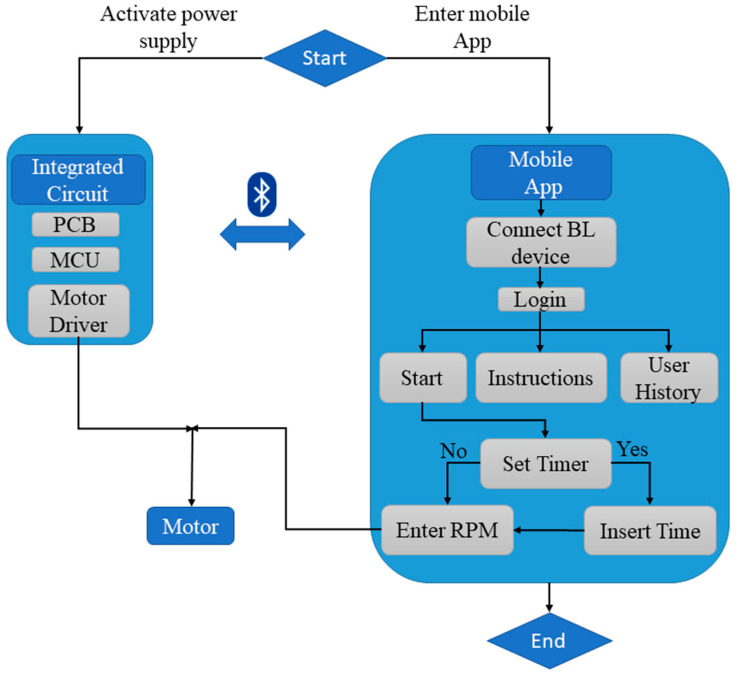
Architectural diagram of the 3D printed IoT-based Bioreactor.

This study aims to bridge this gap by developing a robust do-it-yourself (DIY) bioreactor that utilizes the advantages of additive manufacturing and IoT technology, such as wireless control of the proposed device, real-time data collection, and automating the use of the device in order to save users’ time. The proposed novel device is 3D printed and is based on IoT technologies in order to be wirelessly controlled via Bluetooth protocol. Additionally, an integrated circuit consisting of a microcontroller unit (MCU) is developed using printed circuit board (PCB) design techniques to meet the required electronic hardware specifications. A user-friendly mobile application for Android smartphones was created for better user experience at the experimental stage, and was developed to provide control over the 3D printed IoT bioreactor. The present study utilizes *E. coli* bacteria, conducted with successful proof of concept, and provides results indicating that the bacterial cell culture proliferated, in accordance with the findings of corresponding publications [20,24]. As similar DIY bioreactors have been employed for the cultivation of human cells in suspension, the proposed bioreactor aims to be leveraged with a specific focus on pluripotent stem cells (hPSCs). The architectural diagram of the smart 3D printed IoT-based bioreactor (Figure 1) illustrates its novel features, including the development of a fully functional prototype using 3D printing techniques, which minimized development time and enabled an easily assembled design. The bioreactor was successfully implemented for bacterial growth experiments. Furthermore, potential future applications in the generation of human organoids and the cultivation of diverse biological cultures are envisioned. The design and simplified assembly process of the proposed bioreactor make it suitable for a wide range of biological and biomedical research applications.

This paper’s structure outlines a step-by-step description of the development of the overall 3D printed IoT-based bioreactor, both in terms of hardware and software, as follows. Section 2 presents the Materials and Methods of the developed integrated circuit, firmware and graphical user interface (GUI) for the mobile application, as well as the design and additive manufacturing processes for the bioreactor. Section 3, Results and Discussion, states the outcomes of this study, with the presentation of the final prototype design, the testing methods, and a discussion of the bioreactor’s operational data. Finally, the findings of the study are summarized in Section 4, the Conclusions section.

## 2. Materials and Methods

**Figure 2:**
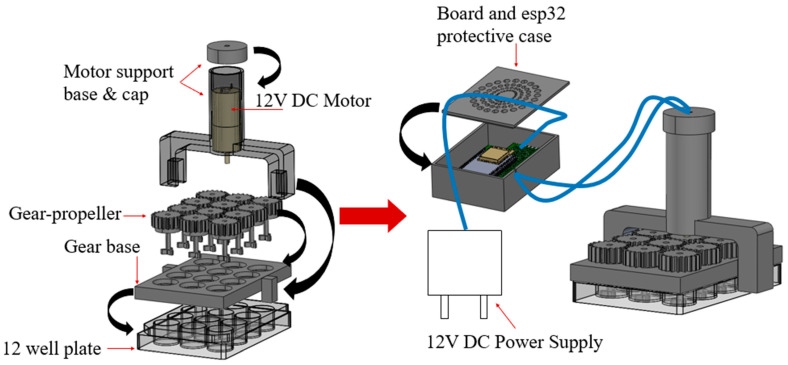
3D Printed Bioreactor.

The objective is the design and manufacture of the four 3D-printed parts that constitute the bioreactor, as they are illustrated in Figure 2. The parts from which the bioreactor is assembled are: (a) the gear-propeller parts (gears with built-in propellers) that transmit the motion generated by the motor, (b) the motor support base which provides the required stability to support the motor, (c) the board and ESP32 protective case, and (d) the gear base where the gear-propeller parts are adapted.

The design of this 3D printed bioreactor has been based on the dimensions of a widely used commercial 12-well plate of the Greiner brand [25]. This helps the easy integration of the bioreactor with the well plate, providing researchers with a convenient and accessible solution for their experiments. The design ensures that the bioreactor fits precisely onto the well plate, and the placement is simple.

### 2.1. Additive Manufacturing of Bioreactor Parts

In this study, the 3D printed parts of the bioreactor, including the gear base, gear-propeller and motor support base, were designed based on the structure of a standard commercial 12-well plate to ensure compatibility. The design of the remaining 3D parts of the bioreactor, such as the board and ESP32 protective case and motor support base cap, took into account the dimensions of the PCB, the electrical cables and the motor. All the printed parts were 3D designed using SOLIDWORKS^®^ CAD Software (2022 SP2.0 Professional version) and fabricated via fused filament fabrication (FFF) using a Prusa 3D printer (Original Prusa MINI+) [26]. The fused filament fabrication technique was chosen due to its widespread adoption, low cost, and easy material accessibility [27].

**Figure 3:**
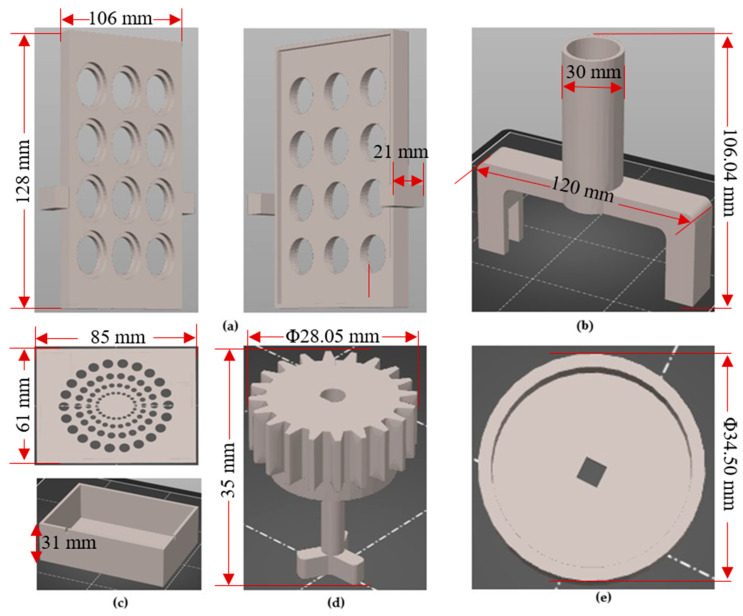
(**a**) Gear base design; (**b**) Motor support base design; (**c**) Electronics housing; (**d**) Gear-propeller design; (**e**) Cap for protecting the motor.

To adjust the 3D printing parameters and generate the G-code, Prusa Slicer 2.5.0 was utilized. The printing parameters were configured as follows: the nozzle temperature was set to 260 °C, the bed temperature was set to 100 °C, the layer height was set to 0.25 mm to determine the print quality, and the infill of the parts was set to 20%. Additionally, a nozzle with a diameter of 0.4 mm was employed for all the printed parts. In terms of the printing material, a 1.75 mm-diameter acrylonitrile butadiene styrene (ABS) filament was utilized during the fused deposition modelling (FDM) process. ABS is compatible with EtO (ethylene oxide) sterilization, but is not compatible with autoclave or gamma sterilization. However, due to its temperature resistance [28], sufficient mechanical properties, toughness and durability [29], ABS exhibits favorable behavior in sterilization methods involving washes in 10% bleach, 70% ethanol, distilled water rinses, and short periods of time in UV irradiation [13,30]. Figure 3 presents the standard triangle language (STL) files of the bioreactor’s components.

Table 1 presents the 3D printed part dimensions, the quantity of each part per bioreactor system and the information used to calculate the total cost. For the calculation of the cost of each part, the price of 1 kWh was considered equal to EUR 0.39, and the average power consumption at 26 °C room temperature is 120 W when printing generic ABS filament [31]. The calculations were based on the average cost of ABS and filament, which was purchased at a price of EUR 29.75 per kilogram (kg) without VAT. Specifically, for the 3D printing of the bioreactor parts, Raise3D Premium ABS filament was used. The calculations for the cost of energy, cost of material and unit cost were conducted using the equations provided below:

(1) $$\mathrm{Cost}\;\mathrm{of}\;\mathrm{material}=\frac{\mathrm{Filament}\;\mathrm{price}\;\left(1000g\right)\times \;\mathrm{Used}\;\mathrm{filament}\;\left(\mathrm{for}\;\mathrm{each}\;\mathrm{part}\right)}{1000}$$

(2) $$\mathrm{Cost}\;\mathrm{of}\;\mathrm{energy}=E\times \mathrm{Price}\;\mathrm{of}\;1\mathrm{kWh}\;\left(0.39€\right)$$

(3) $$E=\frac{P\left(W\right)\times t\left[\left(\frac{\min}{60}\right)+h\right]}{1000\frac{W}{\mathrm{kW}}}$$

(4) Unit cost=Cost of material+Cost of energy

The quantity of filament used in grams and the printing time was obtained from Prusa Slicer after the slicing process of the STL files. The results of these calculation equations for each object are presented in the corresponding cells of the following table.

According to the costs listed in Table 1 for each 3D printed part, the total cost for fabricating one bioreactor amounts to EUR 6.86. This total cost includes the combined expenses for energy and materials. This cost is an estimate and may vary depending on factors such as the price of 1 kWh, the average power consumption of the 3D printer, the cost of the filament and the specific 3D printing parameters (e.g., infill, supports, etc.).

Furthermore, according to the data provided in the table above, it can be concluded that the weight of the bioreactor, excluding the Greiner 12-well plate, is 200.72 g.

### 2.2. Electronic Hardware Design and Development

**Figure 4:**
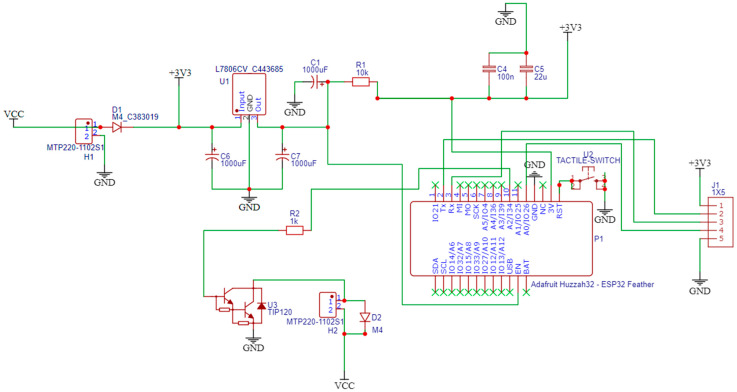
Schematic design.

**Figure 5:**
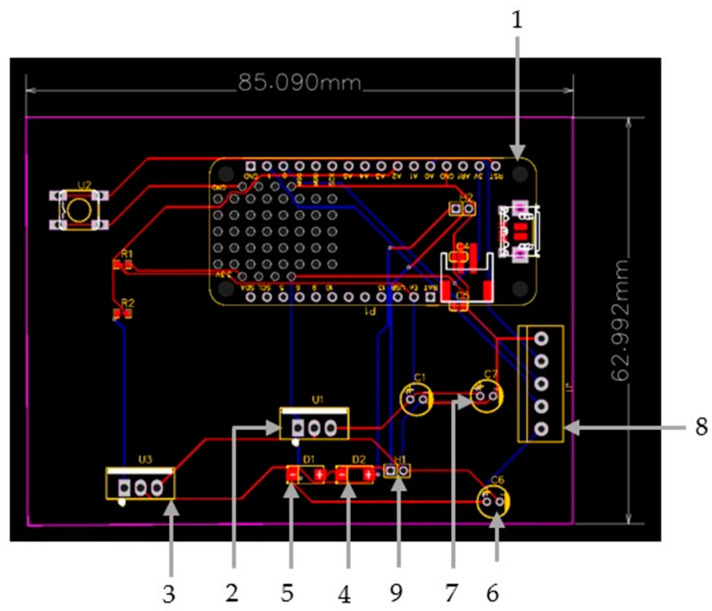
PCB Layout including (1) Adafruit Feather Huzzah ESP32 development board, (2) 7805-voltage regulator, (3) TIP120 NPN Darlington power transistor, (4,5,6,7) diodes, (8) Female headers and (9) terminal blocks.

The electronic hardware design was implemented using the EasyEDA STD (V.6.5.34, 2023) free open-source software. The schematic design of the hardware components is created via EasyEDA, as presented in Figure 4. The PCB layout is exported automatically from EasyEDA, which is shown in Figure 5, with the assembled electronic components being numbered as explained later.

The design is based on a custom PCB that integrates a microprocessor with Wi-Fi and Bluetooth connectivity to control the speed through an application, a motor which integrates a gearbox to lower the maximum speed of the motor to 108 RPM, and other components such as a voltage regulator and an NPN power transistor which drives the motor, as well as filtering capacitors and diodes. Special focus is given to interfacing the different components and identifying unwanted interactions.

In particular, the development of the bioreactor’s electronic system is based on an Adafruit Feather Huzzah ESP32 development board (1), introducing an ESP32 microcontroller. The custom PCB is used to provide the microcontroller with a 5V power source to control the 12V DC motor that is used to move the gears. The system is powered by a 12VDC/1A power supply. A 7805-voltage regulator (2) is used to bring voltage down to 5V and supply the microcontroller. A TIP120 NPN Darlington power transistor (3), controlled by ESP32 MCU via a PWM signal, is used to drive the motor. A diode (4) is connected in parallel with the motor to absorb voltage spikes caused by the motor’s inductors to protect the NPN transistor when it is turned off. An extra diode (5) is used as reverse polarity protection at the system power input. Two electrolytic capacitors (6), (7) are connected to the power supply circuit to provide constant voltage and filter any electrical noise. Female headers (8) are added for connecting the ESP32 board, and terminal blocks (9) are added to connect the 12V power supply and the motor to the system. All these components are carefully soldered to ensure a secure and stable connection on the custom PCB. Finally, in order to confirm that all of the components were properly connected, a multimeter was used to measure and verify stable connections.

The bioreactor’s electronic system for its development is based on a low hardware bill for materials, as is shown in detail in Table 2, with a total cost of EUR 43.60 without VAT. The developed PCB will be packaged inside 3D printed housing during the prototyping stage.

### 2.3. Prototyping of Bioreactor Device

The first step in assembling the bioreactor is placing the gear base above the commercial well plate. The gear base is designed to have slightly larger dimensions than the well plate, in order to fit above it and to be easy for the user to adjust and detach. Then the DC motor is placed inside the motor support base part. One of the gears is attached to the bottom of the motor and then the remaining gears, along with the motor support base, are placed on the gear base part. Finally, the top of the motor support base part is secured with a cap that has a small hole, through which the cables that connect the motor with the PCB pass. The motor support base is a critical component of the overall system design, as it houses the DC motor. The motor support base is designed to be sturdy and durable, providing a stable foundation for the motor to operate smoothly and efficiently. The PCB is housed inside the protective case, which has larger dimensions than the PCB and is designed to have two small gaps: one gap for the cables that connect it with the motor and one for the cables that connect it to the power supply.

The continuous motion of the content of each well is caused by the gear-propeller parts. In a robust architecture, the twelve 3D-printed gear-propellers are connected to each other through an arrangement of gears which are driven by a motor. The design of the propeller consists of three blades placed at equal distances between them and the shaft which has the gear on one side and the blades of the propeller on the other. The propeller is designed to be at the end of the shaft so that there is the possibility of stirring small amounts of culture medium in the well plates. Regarding the protection of the board, the 3D printed case is designed with a pattern of holes so that there is proper ventilation and it does not overheat from continuous use.

The prototype can be divided into two parts: the first part consists of the commercial well plate, the gear base, the gear-propellers and the motor support base and cap with the motor inside it, and the second part consists of the board and ESP32 protective case with the PCB inside it. These two parts are connected with two 1.5 m cables that connect the motor with the PCB. Since the parts are not screwed together with bolts, the device can be easily disassembled at any time, if any of the components needs replacing. The prototype has been designed to be dimensionally compatible with a CO_2_ incubator, facilitating its seamless integration into the laboratory setting. The cables of the power source pass through an opening of the CO_2_ incubator.

The total cost of the customized bioreactor proposed in this study amounts to EUR 50.46, while the total weight is approximately 300 g.

### 2.4. Firmware Development

**Figure 6:**
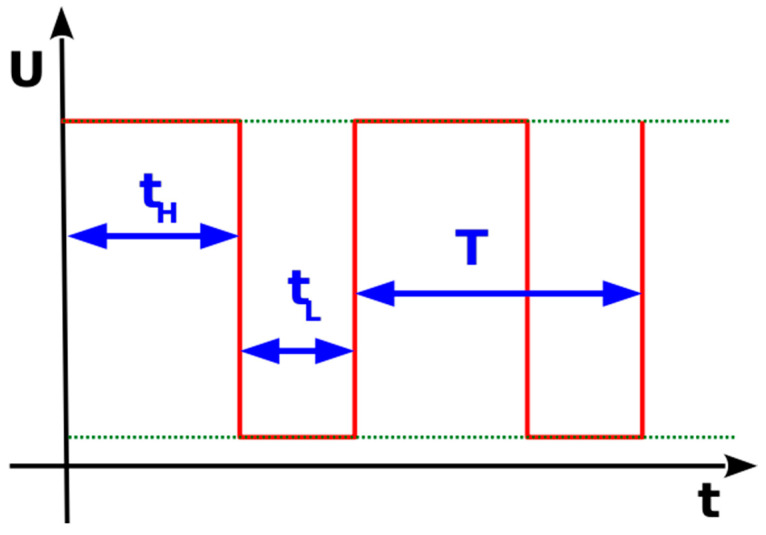
Pulse-width modulation signal.

The firmware related to the control of the 3D printed bioreactor subunits and the interfacing between them was developed and tested with appropriate kits, and it was transferred to the developed hardware. The motor speed is controlled by the generation of a pulse-width modulation (PWM) signal to the base of the NPN transistor. Pulse-width signals are square pulses with controlled ON and OFF states. A PWM signal generated to control analog averages usually has a set frequency (the period is equal to the turn-on time plus the turn-off time) and a variable duty cycle which is produced by regulating the ON (t_H_) and OFF (t_L_) states of the pulse, as is shown in Figure 6. PWM, along with a low-pass filter, is a common method of generating an average analog DC voltage equal to:

(5) $$\;\bar{V}\mathrm{dc}=\sqrt{\mathrm{Vrms}}$$

In this case there is no need for an additional filter, since the coil of the dc motor behaves like a filter itself. The signal generated in this application has a fixed modulation frequency of 100 kHz, while the duty cycle is set to a range from 0 out of 255 (a stopped motor at 0 RPM) to 255 out of 255 (a full speed of 108 RPM). The experimental method will be coordinated with the hardware, simulations, and system diagnostics.

In order to control the DC Motor, ESP32 was programmed using the Arduino IDE software (V.2.1.1). To remote control the bioreactor via a smartphone, Bluetooth communication is established between the microcontroller and the smartphone. Furthermore, the input from the developed mobile application is received and the speed of the DC motor is controlled accordingly. The PWM pulses control the power fed to the transistor, resulting in different base motor speeds and finally controlling the revolutions per minute (rpm) of the motor.

### 2.5. Mobile Application

To facilitate easy referencing of the application within this study and enhance the visual appeal of the developed Android mobile app’s interface, the application is defined as “BioReactor”. Moreover, a logo was created, incorporating the name and an icon representing the bioreactor device. The icon featured in the logo is an illustration representing the CAD design of the bioreactor.

**Figure 7:**
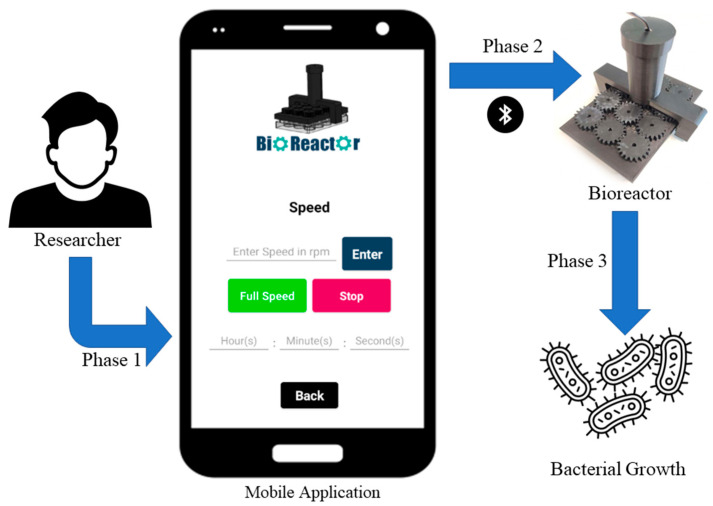
Flowchart of the usage of the Bioreactor.

The important role and the utilization of the mobile application in the bacterial growth process using the bioreactor are explained in three phases, which are depicted in Figure 7. These three phases demonstrate how the potential user has complete control over the entire process of bacterial growth, through the bioreactor.

In particular, Phase 1 involves the use of a mobile application with a user-friendly interface. Researchers can create an account, sign up, log in, and adjust the speed and duration of the bioreactor’s operation through the application. In Phase 2, this application is connected with the bioreactor via Bluetooth and the propellers start rotating at the speed set by the researcher in the mobile application. By utilizing a mobile application, the researcher is able to make real-time changes to the speed of the bioreactor, allowing for precise control of organoid development. Additionally, in this phase, the researcher can determine how many hours the bioreactor will operate continuously. Finally, in Phase 3, the researcher can stop the operation of the bioreactor and examine the results of the process.

To make the stirring process more efficient, a robust Android application with a user-friendly interface was developed to control the functions of the bioreactor. It was developed using the MIT App inventor website (https://appinventor.mit.edu/ (accessed on 20 September 2023)). It also provides a history of users who logged into the application. The application was designed to control the speed of the DC motor that is connected to the ESP32 microcontroller of the bioreactor via Bluetooth communication. It allows users to adjust the speed of the motor in real time, using a fairly simple user interface. Before starting the application, users have to pair their Android phone with the microcontroller of the bioreactor by selecting the device with the name “3DBioReact” and pressing connect.

**Figure 8:**
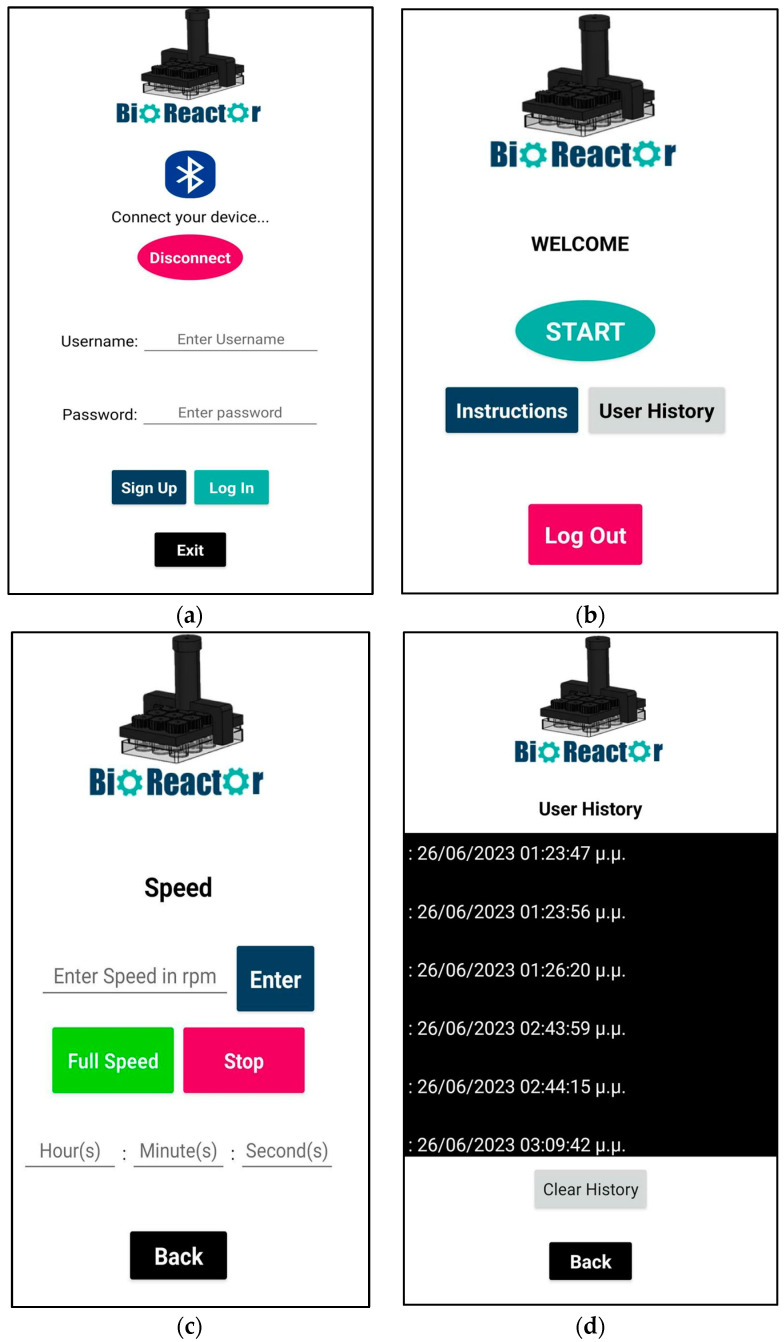
(**a**) Screen when launching the app, with Bluetooth button to connect with bioreactor and buttons to create a new user or to login with credentials; (**b**) Main menu screen; (**c**) Motor speed screen to control the motor with or without timer (**d**) User History screen to keep track of whoever used the app in the specific device that the app is installed in.

When starting the application, the main screen is viewed (Figure 8a). The main screen includes the Bluetooth icon button, which is used to establish a Bluetooth connection between the bioreactor device and the application. Initially, users have to sign up in order to use the features of the application, so that unauthorized access to the motor control system is prevented. The login credentials are stored in the Firebase Realtime Database (https://firebase.google.com/ (accessed on 20 September 2023)).

Figure 8b shows the screen after logging into the application. The main menu consists of three buttons: Instructions, Start and User History. The User History screen shows both the date and time that the user logged in (Figure 8d). Finally, clicking back will return users to the main menu. Clicking the Log out button returns users to the login screen.

The main feature of the application, which can be accessed by clicking the Start button in the main menu, is the DC motor speed control, which allows users to adjust the speed of the motor between 11 and 108 rpm (Figure 8c). The reason for using a minimum of 11 rpm is to ensure that the motor has enough power to spin the gears. Additionally, a reverse timer has been set up which allows users to use the motor for a specific amount of time. When the timer reaches zero, the motor stops.

Overall, the Android application is designed to be user-friendly, with a simple and intuitive interface. The speed control textbox is prominently displayed, making it easy for users to adjust the speed of the motor as needed. The Bluetooth Devices button is easy to find and available on all screens, making it simple for users to establish a Bluetooth connection with the ESP32, even after logging in. Finally, the application is also designed to be reliable and stable, with minimal bugs. The validation test of the mobile app, as well as the bioreactor’s Bluetooth range, included operation in different rooms from that of the proposed device, at a maximum range of 10 m.

### 2.6. Bacteria Population Growth in Smart Biorector Set-Up

**Figure 9:**
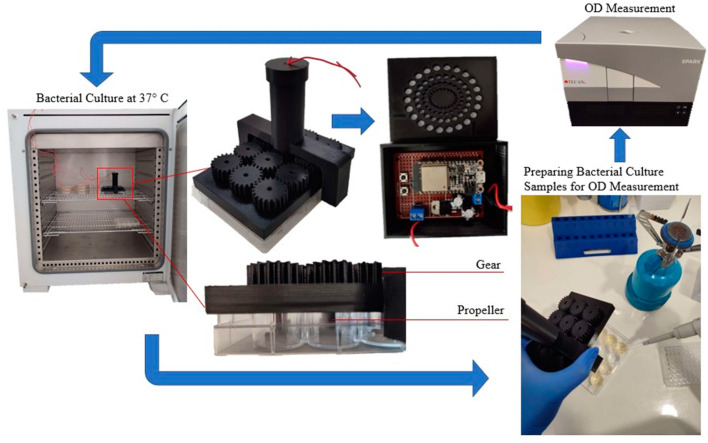
Experimental Setup of the Bioreactor.

*E. coli* bacteria resistant to Ampicillin were cultured overnight in 5 mL of LB medium supplemented with 100 µg/mL Ampicillin at 37 °C. To prevent contamination, an *E. coli* strain transformed with an Ampicillin resistance gene was employed. All cultures were conducted in LB medium supplemented with 100 μg/mL Ampicillin. To ensure sterility during sampling, cultures were handled under aseptic conditions using a Bunsen burner. Bacterial growth was estimated by measuring the OD at 600 nm using a Spark microplate reader (TECAN). The overnight culture was diluted at a ratio of 1:20 to obtain a starting OD of 0.01. Subsequently, 2 mL of the diluted culture was dispensed into each well of a twelve-well plate. Within the twelve-well plate, six wells were filled with bacteria and LB medium and three wells were filled with water, while the remaining three wells were filled solely with LB medium. Wells containing an equal amount of LB medium were used for background subtraction. The plate was incubated at 37 °C using Venticell incubator with stirring at 100 rpm using the bioreactor. A control plate without stirring was incubated in parallel. OD_600_ corresponding to bacterial growth was measured at various timepoints for 48 h. All experiments were performed in triplicates and results are shown as mean ± standard deviation. Statistical significance was calculated using a Student’s *t*-test (Basic protocol 3, [32]). Figure 9 illustrates the aforementioned process that was conducted at various time points to validate bacterial growth.

**Figure 10:**
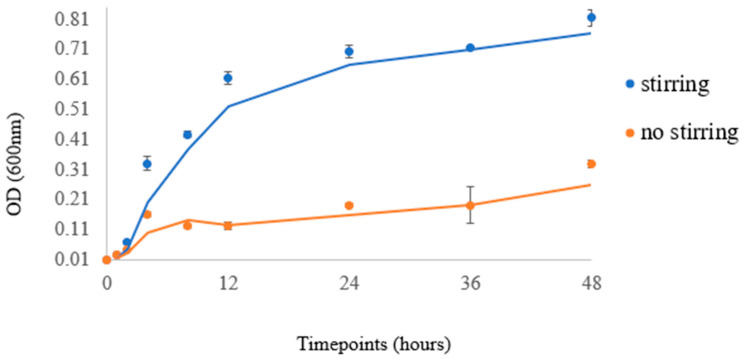
Data points correspond to the readings at 600 nm, while curves indicate the trendline of each experiment.

The cell culture experiments were conducted with multiple replicates. In detail, two independent experiments with six technical replicates of *E. coli* cultures were performed (with a 100% success rate) for sampling at the specified time-points. This is justified by the minimal STDEV per time-point in Figure 10. Sampling was discontinued after 48 h, due to culture saturation (minimal bacterial growth) and the initiation of bacterial decline.

Laboratory strains can grow rapidly in liquid or solid rich media (such as LB medium) in the presence of oxygen at 37 °C, with a doubling time of approximately 20 min. Most strains have been transformed with plasmids encoding antibiotic-resistance genes in order to limit the growth of unwanted products. The growth of bacteria can be modelled with four different phases: a lag phase, exponential phase, stationary phase and death phase. During the lag phase, bacteria adapt themselves to growth conditions and are not yet able to divide. The exponential phase is characterized by cell doubling and high metabolic activity. During the stationary phase, bacterial growth rate and death rate are equal, which is due to a growth-limiting factor such as the depletion of an essential nutrient. Lastly, at the death phase, bacteria start to die. In order to evaluate bacterial growth, optical density (OD) is measured at the wavelength of 600 nm. OD measures the degree of light which is scattered by the bacteria within a culture, and thus OD is proportional to the bacterial population [32].

## 3. Results and Discussion

To assess the bioreactor’s stirring efficiency without interruptions, operational tests were conducted using various smartphones within a laboratory environment. Thorough testing across different devices demonstrated smooth and uninterrupted operation. The evaluation encompassed ten different Android smartphones from various manufacturers. The testing distances were in the range of 10 m in different rooms from that of the proposed device and the control of the device was achieved. Additionally, the test aimed to validate the proper communication between the application and the bioreactor within the incubator at 37 °C, ensuring the absence of malfunctions during future experiments.

According to the methodology, which is presented in the Materials and Methods section, the extracted experimental data of the *E. coli* bacteria (Mach1 strain) at a temperature of 37 °C are presented in Figure 10, which includes the data points that represent the actual readings at 600 nm and the curve corresponding to the trendline of each experiment. The data also demonstrate the various timepoints of the experimental sampling.

An increase in the bacterial population was observed in both stirring and non-stirring conditions. Bacterial cells were in lag phase during the first hours of culture but grew exponentially after 4 h of incubation. The rate of growth in the stirring condition was significantly greater than that of the respective control (non-stirring), especially after 12 h of culture. Stirring strongly induced bacterial growth in a statistically significant manner up to 48 h of culture (*p*-value < 0.001). The growth curve observed in our experiment, when compared with growth curves of *E. coli* reported in the literature [27,28], confirms that the specific bioreactor can serve as a reliable method for bacterial growth in various laboratory settings and applications. These results indicate the value of the bioreactor for culturing cells under stirring conditions, as it emulates the role of a shaking operator that is already used for bacterial cultures. Shaking of the culture helps to control the temperature and aeration of the bacterial growth that affect growth patterns. By agitating the liquid medium, areas of high- or low-nutrient concentration are mixed, and thus shaking causes a decreased lag phase and higher final yield in *E. coli* [33]. This bioreactor creates optimal conditions for the cultivation and proliferation of bacteria and can find applications across diverse fields, including biotechnology and pharmaceuticals. It can also help to maximize bacterial growth, ultimately leading to enhanced productivity in terms of product generation or furthering scientific investigations and research.

## 4. Conclusions

In the present work, the designed smart 3D printed bioreactor has yielded promising results in the proliferation of *E. coli* bacteria. The bioreactor uses an ESP32 and a DC motor, which are controlled by an Android app. The device has proven to be efficient and more cost-effective than traditional bioreactors. Additionally, the 3D printed parts that constitute the device offer a lightweight and easy assembly, ensuring a user-friendly experience for individuals with varying technical expertise. Evaluation of the results indicates that the bioreactor has the capability to adjust and control the stirring rate of the bacteria, which is crucial in maintaining a stable and suitable bacterial-growth-culture environment. The robust performance demonstrated with *E. coli* suggests the proposed bioreactor’s potential for use with other bacterial types.

Collectively, we envision that the proposed DIY, IoT-enabled, multi-well device format could significantly expedite the broader research community’s adoption of 3D hPSC culture expansion and downstream differentiation protocols, such as organoid development.

Future work also involves integrating sensors for real-time monitoring of cell culture parameters like temperature, oxygen levels, pH, and glucose to gather comprehensive data. Additionally, the integration of Wi-Fi connectivity could enable remote interaction with devices located at a distance. However, further studies are needed to understand the full range of potential applications and limitations associated with the proposed bioreactor. Such studies would enable further improvements and the customization of the device to meet the diverse needs of researchers, in order to enhance its functionality.

## Figures and Tables

**Table 1:** Cost analysis of Additive Manufactured bioreactor parts.

Quantity (Items)	Part Name	Dimensions	Used Filament (g)	Printing Time	Cost of Energy (EUR)	Cost of Material (EUR)	Unit Cost (EUR)
12	Gear-propeller	Φ28.05 mm × 35 mm	4.51 × 12 parts = 54.12	35 min × 12 parts = 420 min	0.327	1.61	1.93
1	Motor support base and cap (2 parts)	120 × 30 × 108.04 mm (Length × Width × Height)	47.90	4 h 28 min	0.209	1.42	1.63
1	Board and ESP32 protective case (2 parts)	85 × 61 × 31 mm (Length × Width × Height)	39.08	2 h 45 min	0.128	1.16	1.29
1	Gear base	128 × 106 × 21 mm (Length × Width × Height)	59.62	5 h 6 min	0.238	1.77	2.01

**Table 2:** Hardware Bill of Materials (BOM).

Quantity (Items)	Part Name	Part Number	Unit Cost (EUR)
1	JGA25-370 12 V DC Motor—108 RPM	1140.266	9.03
1	L7806 Voltage Regulator 6 V/1.5 A	L7806CV	0.32
1	Adafruit Feather HUZZAH32—ESP32	ADA3405	20.08
1	Transistor Darlington NPN 5 A—TIP120	TIP120	0.40
1	Resistor ½ W Carbon 5% 1 Kohm	CF1/2W-1K	0.20 (Stack of 10)
2	Diode Rectifier—1 A 400 V	1N4004	0.15 (Stack of 5)
2	Electrolytic Capacitor 16 V 1000 μF	RXJ102M1CBK-1320	0.12
1	12 V AC Adapter	MU18-2120150-C5 K21	13.30
Total Cost			43.60

## Data Availability

Data available on request.
